# Comparing Prophylactic Versus Threshold-Based Insecticide Programs for Striped Cucumber Beetle (Coleoptera: Chrysomelidae) Management in Watermelon

**DOI:** 10.1093/jee/toz346

**Published:** 2020-01-05

**Authors:** John J Ternest, L L Ingwell, R E Foster, I Kaplan

**Affiliations:** 1 Department of Entomology, Purdue University, West Lafayette, IN; 2 Entomology and Nematology Department, University of Florida, Gainesville, FL

**Keywords:** integrated pest management, insecticide, scouting, economics, watermelon

## Abstract

In cucurbit crops such as watermelon, implementation of integrated pest management (IPM) is important due to the high reliance on bees for fruit set, along with mounting evidence of the risks of insecticide use associated with pollinator health. Yet, IPM adoption, on-farm pesticide use behaviors, their costs, and impacts on the primary insect pest (striped cucumber beetle, *Acalymma vittatum* F.) are poorly known in one of the key watermelon-growing regions, the Midwestern United States. To better understand how to implement IPM into watermelon production, we assessed pest management practices on commercial watermelon farms using 30 field sites in Indiana and Illinois over 2 yr in 2017 and 2018. Across all sampling dates, beetles never crossed the economic threshold of five beetles/plant at any farm and most were maintained at densities far below this level (i.e., <1 beetle/plant). Moreover, we documented a wide range of insecticide inputs (mean ca. 5 applications per field per season; max. 10 applications) that were largely dominated by inexpensive foliar pyrethroid sprays; however, insecticide application frequency was poorly correlated with pest counts, suggesting that most of these applications were unnecessary. We calculated that the cost of the average insecticide program far exceeds the cost of scouting, and thus IPM is estimated to save growers ca. $1,000 per field under average conditions (i.e., field size, insecticide cost). These data strongly indicate that current management practices on commercial farms in the Midwest would benefit from implementing more threshold-based IPM programs with potential increases in both farm profitability and pollination services.

A variety of complex sociological factors underlie the decision-making process used by farmers in managing pests, e.g., whether and when to apply insecticides to their crop (Baumgart-Getz et al. 2012, Jabbour et al. 2014, Chouinard et al. 2015, Magarey et al. 2019). The obvious drawback of underestimating pest threat is a loss in yield and, correspondingly, profit. Because the consequences of underestimation can threaten farm sustainability, many farmers select a more aggressive—and overly conservative—management approach that reduces or eliminates the perceived risk posed by insects. For example, a recent analysis of 946 conventional (i.e., nonorganic) farms suggested that total insecticide use could be reduced by 60% without losses in productivity or profitability (Lechenet et al. 2017). This conservative approach that overuses insecticides comes with its own set of unique challenges including, the evolution of resistance in the target species (Gould et al. 2018), disruption of beneficial insect populations (Desneux et al. 2007, Douglas and Tooker 2016), and economic losses in cases where costly insecticides are applied over large acreages without sufficient pest pressure to justify the expense (Krupke et al. 2017a,b).

In theory, these two opposing forces should result in stabilizing selection for an intermediate management style that balances the beneficial and detrimental aspects of insecticide use. Integrated pest management (IPM) was designed as a holistic tool by which growers can achieve this balance (Stern et al. 1959, Smith 1962, Geier 1966, Pedigo and Rice 2009). Despite being a cornerstone of sustainable agriculture for decades, the topic has gained renewed interest in recent years as IPM principles appear to have eroded in certain crops, leading some to question ‘Whatever happened to IPM?’ (Peterson et al. 2018). This resurgence is also fueled by growing concern over the potential contribution of pesticides to declines in pollinator health (Potts et al. 2010, Goulson et al. 2015a,b), especially in pollination-dependent crops where bees strongly contribute to yield (Biddinger and Rajotte 2015, Mallinger et al. 2015, Stanley et al. 2015, Vaughan et al. 2017).

Cucurbits, for example, are among the most highly pollinator reliant crops, yet also suffer from persistent attack by a diversity of economically damaging arthropods, resulting in conflicts over how to effectively control pests without disrupting pollination. In our study area—the Midwestern United States—the primary pest of concern is the striped cucumber beetle (SCB), *Acalymma vittatum* (F.). SCB cause direct damage to plants, chewing roots as larvae and leaves as adults, over two generations each growing season (Gould 1943, Foster and Brust 1995). In addition, SCB vector the devastating bacterium *Erwinia tracheiphila*, which causes bacterial wilt that kills infected plants (Brust 1997, Rojas et al. 2015). As a result, tolerance for SCB is extremely low in some cucurbits, i.e., economic threshold of 1 beetle per plant in cucumber and muskmelon (Brust and Foster 1999). Watermelon, on the other hand, is not susceptible to bacterial wilt and thus the economic threshold was set at the much higher five beetles per plant (Foster 2016). Despite these established thresholds, anecdotal observations and grower surveys indicate that insecticides are applied far more regularly than needed. In surveys conducted at several grower-attended extension meetings during the winter of 2017 (R.E. Foster, unpublished data), none of the participants used the above-cited thresholds when asked to describe their SCB management practices. Most claim to apply foliar insecticides either ‘when I see live beetles’ or ‘on a regular basis or when I apply fungicides’.

Although these precautionary treatments are likely viewed as low cost insurance, they increase the potential for yield drag due to suboptimal pollination. Most watermelon production is triploid (seedless), requiring bees to move pollen across several rows from diploid donors (aka pollenizers). Producers typically rent managed honey bee (*Apis mellifera*) hives to provide the 16–24 visits per flower needed for maximum pollination (Walters 2005). Unmanaged, wild bees also contribute to fruit set (Kremen et al. 2002, Winfree et al. 2008, Rader et al. 2013). Several studies now show that systemic neonicotinoids applied to the soil for SCB control are detectable in cucurbit nectar and pollen at levels shown to be harmful for bees (Dively and Kamel 2012, Stoner and Eitzer 2012, Nixon 2014). Further, when experimentally compared, threshold-based insecticide applications after scouting for SCB resulted in higher muskmelon yield and economic returns than calendar-based insecticide applications, which the authors attribute to pollination differences (Brust and Foster 1995, Brust et al. 1996). These data from small-scale research plots collectively indicate that the type of insecticide used (i.e., systemic vs contact) and application frequency are critical factors in cucurbit pest management; however, it is unknown how often commercial growers apply insecticides on-farm, which types they use, how these applications affect SCB populations, and whether their overall program fits an IPM framework.

In this study, we conducted a 2-yr survey of insecticide use and SCB abundance on commercial watermelon farms in Indiana, typically one of the top five or six watermelon producing states in the United States and Illinois (USDA-NASS 2017). We used these data to quantify SCB aggregation and calculated optimal sample sizes for assessing field densities. SCB are notorious for aggregating on plants, resulting in a patchy distribution across fields, making them difficult to scout, i.e., how many plant samples are needed to provide a reliable population estimate? (Foster 1986, Ferguson et al. 2003). We suspected this knowledge gap is a factor inhibiting the adoption of IPM in this system. Further, we compared the cost of an IPM-based scouting plan using thresholds with current insecticide inputs employed by commercial farmers to determine the cost savings associated with transitioning to IPM.

## Materials and Methods

### Field Sites

**Fig. 1.**
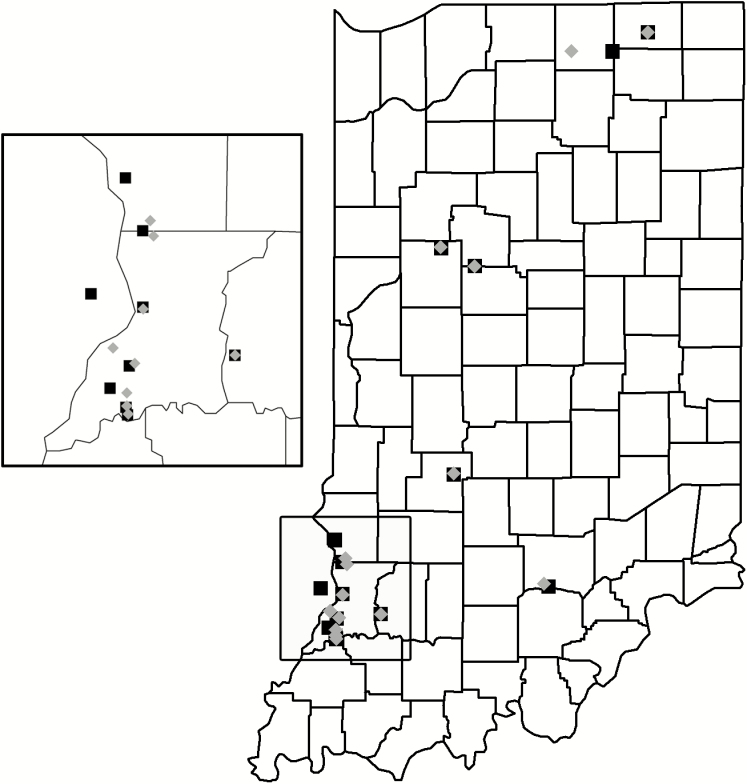
Indiana map with the locations of field sites in 2017 and 2018. Black squares and grey diamonds represent field sites in 2017 and 2018, respectively. Inset on the left is a close-up of Knox and neighboring counties. This is the primary watermelon production region in Indiana and where 18 of the 30 field sites were located. One field was located over the Indiana border in Lawrenceville, IL in 2017.

SCB were sampled in 15 commercial watermelon fields in Indiana and Illinois between 23 May and 16 August 2017 and 15 additional fields in Indiana between 21 May and 14 August 2018 (Fig. 1). Fourteen of the 15 farms were sampled in both years, even though the sampled field location varied between years, whereas two farms were only sampled 1 yr. All references to farm describe the managing farm, not the individual field plot that was sampled. Sampled fields varied in size from 0.365 to 100 acres. Management practices and inputs were determined by the growers, ranging from frequent prophylactic applications of conventional insecticides to organic production (see Table 1 for summary data). Grower-reported pesticide records were collected from 28 of the 30 fields. All insecticides and miticides were included in analyses (i.e., not only SCB-targeted applications). Analyses were duplicated with the exclusion of miticides and all results were qualitatively consistent with the presented results. None of the growers used a formal scouting program for SCB to inform insecticide applications; however, all growers were aware of the threat posed by this pest. A subset used informal scouting (i.e., walking through the field looking for SCB) to guide insecticide decisions based on personal experience rather than the suggested threshold. All farms had managed honey bee hives—and commercial bumblebees were also used in a few cases—in or near the sampled fields.

**Table 1.** Size and description of watermelon fields used in 2017 and 2018

Farm #	Field size (ac) 2017	Field size (ac) 2018	Pest management	Farm type
1	1.78	1.52	Conventional	Diversified
2 (2017 only)	3.29	--	Conventional	Diversified
3 (2018 only)	--	1.32	Conventional	Diversified
4	2.00	6.65	Conventional	Diversified
5	0.686	0.365	Organic	Diversified
6	0.578	4.53	Conventional	Diversified
7	20.5	17.9	Conventional	Diversified
8	100.0	57.2	Conventional	Watermelon
9	22.4	8.84	Conventional	Diversified
10	43.0	7.62	Conventional	Watermelon
11	31.7	54.9	Conventional	Diversified
12	2.50	2.21	Conventional	Diversified
13	15.7	34.1	Conventional	Watermelon
14	28.0	6.77	Conventional	Watermelon
15	13.7	10.6	Conventional	Watermelon
16	29.5	26.5	Conventional	Watermelon

Over the 2 yr, 16 commercial operations were used in the study, with 14 participating both years and two participating for 1 yr each. For farm type, diversified operations were differentiated from primarily watermelon production by the presence of three or more on-farm crops.

### Pest Sampling

Fields were sampled weekly from transplant to initiation of flowering and bi-weekly thereafter. This transition was due to reduced pest pressure and plant vulnerability during this period. In total, each field was sampled between 6 and 12 times, depending on transplant date and weather. Sampling events consisted of walking five transects in 2017 and four transects in 2018. Linear transects were positioned randomly, perpendicular to the field edge, and spaced at least 10 m apart from one another. Transects extended along the plant rows and were alternated between the beginning and end of the row. Plants were sampled at 25, 100, 175, and 250 m along each transect to account for potential variation in beetle counts occurring at the field edge versus the interior. In fields less than 250 m long, the sampled plants were evenly spaced across the entire length of the field. We counted the number of SCB on 20 plants per field during each scouting event in 2017 and 16 plants in 2018. This change in sampling intensity was in response to 2017 data, which indicated that the sample number needed to accurately detect the beetle threshold was lower than expected.

SCB are commonly found inside flowers as well as on leaves, especially the underside of leaves when it is hot. Therefore, at each sampling location, vines were carefully overturned to visually observe the top and underside of each plant, including stems, leaves, and flowers. The soil and plastic mulch were also inspected since beetles often reside beneath the plant. Early in the season, individual plants were easily distinguishable from one another; however, as the vines grew together, a 1-m^2^ area was designated as an individual plant. Best efforts were made to only sample plants destined to produce watermelons and avoid sampling pollinizer plants. Although when sampling a 1-m^2^ area, it can be challenging to differentiate individual plants.

Altogether, we conducted 281 individual farm visits, sampling 5,016 plants across the 2 yr. Beetle counts were averaged per field across the plants sampled during each visit to calculate the mean number of beetles per plant per date. In 130 of the 281 visits, no beetles were observed on any sampled plant. These data were excluded when calculating aggregation and recommended sample size.

### Quantifying Aggregation

Spatial distribution can be measured with the variance to mean ratio of pest counts (Ruesink 1980, Foster 1986). This ratio was used to assess dispersion of SCB across the 151 visits when beetles were observed. The mean beetles per plant (*m*) and variance ($s^{2}$) were used to calculate the variance to mean ratio. When s^2^ = m, the population is assumed to be randomly distributed, while $s^{2}<m$ means the population is uniformly distributed and $s^{2}>\;m$ indicates aggregation (Foster 1986).

Another measure of spatial dispersion introduced by Iwao (1968) uses linear regression on Lloyd’s mean crowding ($m_{c}$) which expresses the mean number of other individuals in the area per individual observed, defined as $m_{c}=m+\left(\left(\frac{s^{2}}{m}\right)-1\right)$, to mean density (Lloyd 1967). This creates a linear relationship with the intercept (*a*) and the slope (*b*) used to assess dispersion. Mean pest density was regressed on mean crowding for each of the 151 visits when beetles were observed. An a>0 and b>1 indicate an aggregated pest distribution, while a=0 and b=1 indicate random distributions.

Taylor’s power law relates sample variance to the sample mean with the expression $s^{2}=a{\bar{x}}^{b}$ where ($\bar{x}$) is the mean and ($s^{2}$) is the variance (Taylor 1961). These variables were calculated, and a linear regression was performed for $\log s^{2}$ on $\log\bar{x}$ using the same 151 visits. The *a* value was quantified by taking the untransformed intercept ($a=10^{intercept}$) while the *b* value was the slope of the regression. These *a* and *b* values were then used to determine the sample size (*n*) needed to scout with the recommended 25% precision (c=0.25) (Foster 1986) using Ruesink’s (1980) equation: $n=\frac{a{\bar{x}}^{b-2}}{c^{2}}$. These calculations were performed to assess SCB aggregation behavior and develop an effective scouting methodology to be used by watermelon growers in the Midwest.

### Economic Analysis

Pesticide records from each field were then used to calculate cost per acre and total cost per field for each insecticide application on each farm, as well as total insecticide costs for the growing season. Insecticide costs were compiled from either direct expenditure reports by growers or prices sourced from the 2019 NDSU Extension Insect Management Guide (Knodel et al. 2018). All prices were based on the product used or the closest comparable product.

To estimate potential costs saved from eliminating a single insecticide application, we used a range of field sizes and insecticide prices based on observed values. Field sizes were selected from the range encountered in the study: 1 acre represents the smallest-scale production modeled; 5 acres was a typical small plot; 20 acres was the average of all fields; 50 acres was a typical large-scale plot; and 100 acres was the largest field in the study. Insecticide expenditures were selected from reported costs of commonly used insecticides: $1.00 per acre is the lowest cost insecticide, a low rate permethrin treatment; $5.00 per acre is approximately the cost of many cheaper insecticides; $9.75 per acre is the average cost of all reported insecticides; $30.00 per acre is approximately the cost of many expensive insecticides; and $57.15 per acre is the highest reported cost insecticide, a high rate chlorantraniliprole treatment.

The ranges of actual grower expenditures on insecticides were compared with costs for implementing a scouting program that employs thresholds, in the case of watermelon, five beetles per plant. The cost of scouting was assumed to be a product of time spent visually searching plants for beetles, the number of plants sampled per field (estimated via Ruesink (1980) in above section), and the price of paying an employee to complete this task. In 2018, we recorded the amount of time to complete a transect of four plants on 505 transects. Hourly pay for scouts were assumed to range from the minimum wage in Indiana of $7.25 per hour to $15.00 per hour, which was based on the average wage rate of $14.29 for all hired farm workers in Indiana for summer 2018 (USDA-NASS 2018).

### Statistical Analysis

Grower pesticide use was categorized as follows. First, the presence or absence of a neonicotinoid or other systemic insecticide pretreatment using transplant soil drench, or insecticide application prior to or at transplant as a prophylactic application. Second, we assessed the number of foliar insecticide applications on the crop over the course of the season. This provides a coarse overview of management intensity, recognizing that the applied chemicals vary widely in their toxicity, rate, and mode of action. Using these data, we separated fields into three management groups: 1) no insecticide treatment (P-F-), 2) no pretreatment but subsequent foliar applications (P-F+), and 3) both a pretreatment and foliar applications (P + F+). No fields used a pretreatment only without subsequent foliar insecticide applications (P + F-).

The maximum SCB density and season-long average densities were analyzed using analysis of variance (ANOVA) with the three management groups as treatments. A Tukey test was used to assess the relationships between groups when ANOVA results were significant based on a significance level of 0.05. The mean and standard error statistics for season-long average and maximum SCB density are reported for each management group. To assess the impact of management intensity as a continuous variable, we used a regression with the number of foliar insecticide applications in the 24 fields that were treated with insecticides as the predictor variable and maximum or seasonal average SCB densities as the response variables.

Pesticide use behaviors were assessed with regression to examine the relationships between 1) field size and insecticide expenditure per acre and 2) insecticide application frequency and insecticide cost. These analyses were used, respectively, to test whether large versus small growers differ in their investment in insecticide-based pest management, and whether cheap versus expensive products are preferentially used by growers.

All analyses were performed using R, version 3.5 with the ‘stats’ package (R Core Team 2018). Figure 1 generated with ‘ggmap’ (Kahle and Wickham 2013), all other figures were generated using the ‘ggplot2’ package (Wickham 2009).

## Results

### Pest Densities, Aggregation, and Sampling

**Fig. 2.**
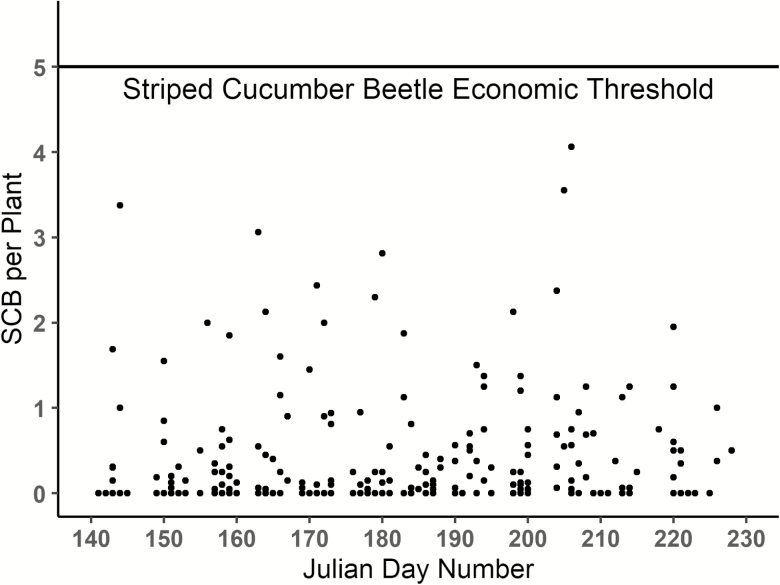
Mean SCB densities per watermelon plant for all 30 field sites across the 2017 and 2018 field seasons. The threshold of 5 SCB per plant is indicated with the horizontal black line. No sampling date in any field during the two seasons reached threshold.

SCB was the only insect pest regularly observed in watermelon fields in either year of the study. However, population densities were consistently well below the threshold of five beetles per plant (global mean across all farms, visits, and years = 0.35 beetles per plant). In fact, the threshold was never reached in any of the 30 fields from 2017 to 2018 across 281 field visits, including the untreated organic fields (Fig. 2). Densities were so low that an average of two or more beetles per plant was only observed during 4% of the visits. Nearly half (130) of all scouting visits over the 2 yr found no SCB at all.

Each of the dispersion indices indicate that SCBs have an aggregated distribution in watermelon fields (Table 2). At the SCB economic threshold of five beetles per plant, sampling eight randomly selected plants will provide an accurate estimate of mean density per field (Table 3). A more intensive sampling strategy that could accurately detect SCB at lower densities is also included in Table 3. This could be an attractive strategy for some growers due to the low relative cost of increased sampling in a high value crop such as watermelon.

**Table 2.** Mean, variance to mean ratio, mean crowding, Iwao’s regression *a* and *b*, and Taylor’s power law *a* and *b* calculations used to assess SCB aggregation in commercial watermelon fields during 2017 and 2018 field seasons

Mean (*m*)	Variance to mean ratio ($s^{2}/m$)	Mean crowding ($m_{c}$)	Iwao’s regression (*a*)	Iwao’s regression (*b*)	Taylor’s Power Law (*a*)	Taylor’s Power Law (*b*)
**0.664**	**1.833**	**1.291**	**0.468**	**1.238**	**1.653**	**1.138**
	**$s^{2}/m$** > 1 indicates aggregated distribution		*a* > 0 indicates aggregated distribution	*b* > 1 indicates aggregated distribution		

**Table 3.** Number of plant samples (rounded to the nearest whole number) necessary to assess a range of mean densities of SCBs with 25% precision

Mean Density ($\bar{x}$) SCB	0.5	1	2	3	4	5	10
Number of Plant (1-m2 plant area) Samples (n)	48	26	15	10	**8**	7	4

Recommended plant samples of eight required to detect approximately four beetles per plant highlighted in light gray.

### Insecticide Impact on SCB

**Fig. 3.**
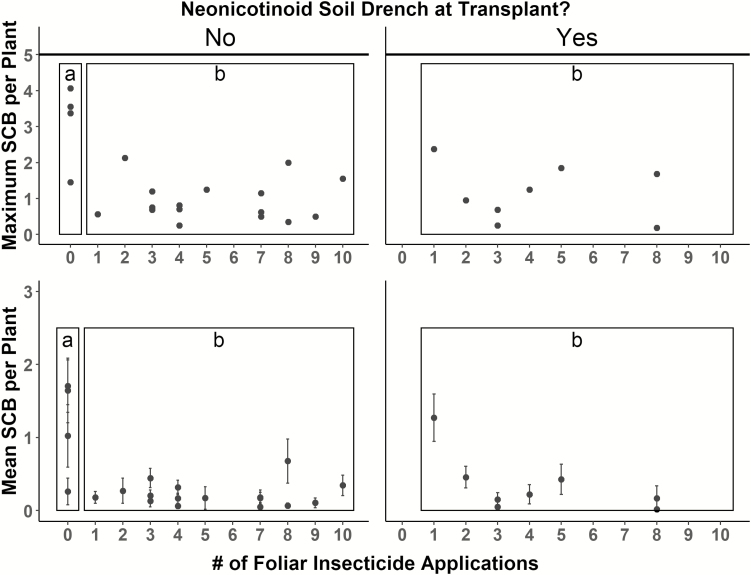
The seasonal maximum (top) and mean (±SE) (bottom) SCB densities per watermelon field based on the number of foliar insecticide applications and the presence or absence of a neonicotinoid soil drench at transplant. The boxes around data points represent the three management groups, from left to right, P-F-, P-F+, and P + F+ The no insecticide treatment (P-F-) group had higher maximum and mean SCB densities than the other management groups (P-F + and P + F+), although threshold was never reached during any visit to any field. Additionally, the number of foliar insecticide applications had no impact on mean or maximum SCB density in the 24 fields that made at least one insecticide application (P-F + and P + F+).

At-planting soil treatment with a systemic insecticide (P) was used in 8 of the fields, 4 fields never used any insecticides, and foliar insecticides (F) ranged from 1 to 10 applications. Management groups (P-F-, P-F+, and P + F+) impacted SCB maximum (*F*_2,25_ = 14.83, *P* < 0.001) and seasonal average densities (*F*_2,25_ = 12.79, *P* < 0.001). More specifically, a post-hoc Tukey test showed the no treatment group (P-F-) had higher SCB max (mean = 3.11 ± 0.57 (SE), *P* < 0.001) and season-long average densities (mean = 1.19 ± 0.34 [SE], *P* < 0.001) than either of the other two groups (P-F + SCB maximum mean = 0.94 ± 0.14 [SE], SCB season-long average mean = 0.22 ± 0.04 [SE]; P + F + SCB maximum mean = 1.15 ± 0.28 [SE], SCB season-long average mean = 0.35 ± 0.14 [SE]), which did not differ from one another (Fig. 3). Treatment group by application frequency interactions were run for both maximum and average SCB densities and neither interaction had a significant effect. Additionally, of the 24 fields that were treated at least once, there was no relationship between number of foliar insecticide applications and SCB maximum (*F*_1,22_ = 0.15, *P* = 0.706) or average densities (*F*_1,22_ = 1.99, *P* = 0.171).

### Economics of SCB Management

Across all farms, an average of 4.5 insecticide applications per field was used during a growing season (see Supp Appendix 1 [online only] for complete insecticide programs per field). However, this includes an organic farm that used zero; thus, the true mean for conventional watermelon farms is ca. 5 per season. Of the 15 active ingredients recorded, the top three most commonly used insecticides were all pyrethroids (Table 4): permethrin (29 applications), bifenthrin (23 applications), lambda-cyhalothrin (18 applications). Neonicotinoids were less commonly used with 4, 5, and 6 applications of thiamethoxam, imidacloprid, and acetamiprid, respectively. The 15 insecticide active ingredients used varied in cost from $0.18/fl. oz. to $8.59/fl. oz., resulting in a range of $1.02/acre to $57.15/acre per spray based on reported application rates (Table 4) (1 fl. oz. = 29.57 ml). The cost of the cheapest insecticides on small fields was as low as $1 per application, while the most expensive treatments on large fields could cost as much as $5,715 per application (Table 5). The average cost of insecticides ($9.75/acre) on an average field size of 20 acres would cost $195 per application.

**Table 4.** The active ingredients used during the 2017 and 2018 watermelon seasons, along with the trade names of those active ingredients

Active ingredient	Trade names	# of applications	Cost/fl. oz. range	Cost/acre range
Chlorantraniliprole	Coragen IC	4	$7.63	$22.89–$57.15
Acetamiprid	Assail	6	$6	$24–$36
Spiromesifen*	Oberon	12	$2.24–$3.49	$17.90–$27.92
Fenpyroximate*	Portal XLO	3	$0.79	$25.35
Flupyradifurone	Sivanto	1	$2.57	$17.99
Abamectin*	Abacus, Agri-Mek, Reaper, Tide Timectin	8	$1.09–$2.11	$7.38–$17.44
Imidacloprid	Advise Four, Malice 2F	5	$0.29–$1.58	$4.64–$16.59
Cyantraniliprole	Verimark IC	1	$7.11	$15.36
Flubendiamide	Belt	1	$8.59	$12.89
Thiamethoxam	Platinum	4	$2.16	$10.80
Cyfluthrin	Tombstone	11	$2.19	$5.48–$6.13
Dimethoate	Dimethoate	1	$0.77	$5.92
Bifenthrin	Bifenthrin, Bifenture EC, Brigade, Sniper	23	$0.66–$0.90	$1.80–$5.76
Lambda-cyhalothrin	Grizzly, L-C, Lambda-Cyhalothrin, Warrior	18	$0.86–$2.47	$1.72–$4.94
Permethrin	Permethrin, Permup, Pounce	29	$0.18–$0.35	$1.02–$2.10

The cost/fl. oz. range based on insecticide expenditures reported by growers or the NDSU Extension Insect Management Guide (Knodel et al. 2018). The cost/acre range was calculated using the cost/fl. oz. multiplied by the fl. oz./acre rate that was used by growers. Active ingredients followed by an asterisk denote miticides. Table organized by maximum reported cost of application for each active ingredient.

**Table 5.** The cost per acre that could be saved by reducing one insecticide application over the course of the season on fields of varying sizes and using active ingredients varying widely in per cost application

Cost per acre	1 acre	5 acres	20 acres	50 acres	100 acres
**$1/acre**	$1	$5	$20	$50	$100
**$5/acre**	$5	$25	$100	$250	$500
**$9.75/acre**	$9.75	$48.75	$195	$487.50	$975
**$30/acre**	$30	$150	$600	$1,500	$3,000
**$57.15/acre**	$57.15	$285.75	$1,143	$2,857.50	$5,715

Both field size and insecticide cost values are within the ranges we recorded for participating farms.

**Fig. 4.**
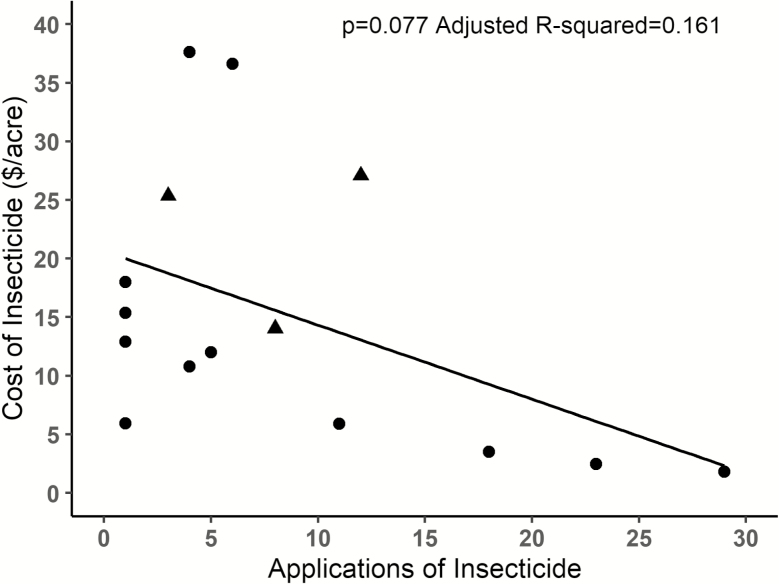
Relationship between the total number of insecticide applications made over the 2-yr study (summed across all fields) and cost of the associated insecticide. Each data point is a different insecticide active ingredient used by growers. Applied miticides are represented by triangles.

**Fig. 5.**
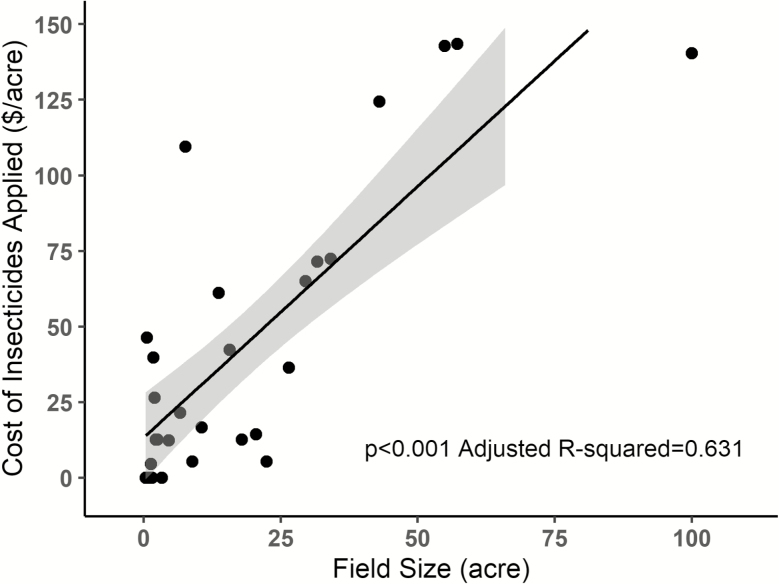
Relationship between field size and total insecticide application costs, standardized on a per acre basis for direct comparison among small and large producers. Insecticide costs may differ due to farm scale and the potential for bulk discounts although we do not expect this to impact the conclusion that larger fields spend more on insecticides. Each data point is one of 28 unique fields sampled. The grey shadow surrounding the best-fit regression line delineates the 95% confidence interval.

Overall, growers tended to more commonly apply cheaper insecticides; there was a negative relationship between application frequency and cost per acre, although this relationship was marginally significant (Fig. 4; *P* = 0.077, adj. r-sq = 0.161). When miticide applications are removed from the analysis the qualitative result remains consistent with marginal significance (*P* = 0.093, adj. r-sq = 0.182). In addition, large growers spent nearly 10 times more than small growers on insecticides when standardized per unit crop area (Fig. 5). This was the case when evaluating the impacts of field size on total cost of insecticides per acre (*P* < 0.001, adj. r-sq = 0.631), average insecticide costs per acre (total cost/number of applications; *P* = 0.027, adj. r-sq = 0.166), and insecticide application number (*P* < 0.001, adj. r-sq = 0.355).

For scouting, the mean time to complete a transect was nearly 9 min. To account for variation among individuals, we considered two sampling times: 10 and 20 min per four plant transect. The 10-min transect is similar to the pace set in our study, while the 20-min transect allows for more leisurely scouting and accounts for greater distances to walk on large fields. Based on the recommended sampling of eight plants per field, scouting at the 10- and 20-min paces could reasonably be completed in 20–40 min. Assuming a weekly scouting plan for the nearly 12-wk watermelon growing season, a total of 4–8 h per field is needed to effectively detect beetles at threshold levels. Using potential labor costs for scouts ranging from the Indiana minimum wage of $7.25–$15.00 an hour, this represents a total expenditure of $29–$120 to scout a field for SCB on a weekly basis across the season, assuming cheap/fast or slow/expensive individuals as the extreme positions, respectively.

## Discussion

Threshold-based monitoring as part of an IPM program generally improves pest control, reduces input costs, increases profitability, and/or reduces nontarget effects on beneficial insects across a diversity of crops including, e.g., pepper (Prager et al. 2016), tomato (Trumble 1998), celery (Trumble 1990), tobacco (Slone and Burrack 2016), and onion (Leach et al. 2017, 2019). Similarly, employing pest thresholds outperforms calendar-based spraying in watermelon when tested in small research plots (Lima et al. 2014), with analogous outcomes in related cucurbits such as muskmelon (Brust et al. 1996). Yet, our on-farm data tracking commercial grower practices indicate that SCB densities consistently fall below the economic threshold of five beetles per plant, regardless of management intensity. Even the few fields that never applied insecticides did not exceed the threshold, and overall there was no relationship, or trend, linking insecticide application frequency—spanning the range from 1 to 10 treatments per field per season—and SCB abundance. These data collectively suggest a discordance between the actual threat posed by SCB in watermelon and current insecticide use by commercial producers.

An alternative explanation is that sprays targeted other non-SCB insects; this is supported by miticide and insecticide use that is not labeled for SCB. It is unclear what caused growers to apply pesticides targeting other pests; however, we expect these are prophylactic responses to mites and non-SCB insects. The miticides used by growers were consistently applied during a period (mid-June) when rye strips used as wind breaks are mowed. When these strips are removed, growers fear that mites could migrate from rye to watermelon and apply a miticide in response (D. Egel, personal communication). The insecticides that were used and not labelled for SCB were likely either targeted for other pests or a result of mistaken pest identity. SCB larvae have been observed feeding on watermelon rinds similar to the lepidopteran pest known as pickleworm*, Diaphania nitidalis* (Lepidoptera: Crambidae). It is possible that growers applied these insecticides to treat for SCB larvae they believed to be pickleworm. Yet, we never observed appreciable densities of secondary pests and therefore conclude that these applications were prophylactic treatments.

Moreover, when experimentally evaluated, insect pest pressure overall is too low and sporadic to justify regular insecticide use in watermelon (Lu et al. 2003, Lima et al. 2014). In Oklahoma, for example, insecticides provided no pest management benefit to watermelon for control of squash bug, *Anasa tristis* (Hemiptera: Coreidae), and thus did not affect yield compared to the untreated control (Dogramaci et al. 2004). It is difficult to extrapolate these results across broad geographic regions, however, where different pest complexes can have relatively greater impacts. In the Southeastern United States and Brazil, for instance, aphids (Webb and Linda 1993), whiteflies (Roberts et al. 2007, Webb et al. 2012, Lima et al. 2018), and thrips (Pereira et al. 2017, Barbosa et al. 2019) can cause substantial direct damage and/or transmit devastating viruses to watermelon. In the Midwestern United States, we tend to only observe these ‘greenhouse pests’ in protected culture (e.g., high tunnels), rarely in open-field production. Spider mites are occasionally a problem, but typically in response to broad-spectrum insecticide applications that disrupt natural enemy populations (Trichilo and Wilson 1993).

### Insecticide Use Behaviors

Given that SCB cannot transmit bacterial wilt to watermelon, their main threat is by overwhelming newly transplanted seedlings. Thus, we expected more growers to employ a pest management tactic whereby systemic neonicotinoids are applied as a root drench during transplant to provide a brief (2–3 wk) window of protection to the vulnerable seedling stage, followed by no subsequent control measures on the larger, more defoliation-tolerant vines. Indeed, in other cucurbits that are direct seeded—namely, cucumber, muskmelon, and pumpkin—a FarMore seed treatment containing thiamethoxam is commonly used (note: this seed treatment is unavailable in watermelon). Yet, interestingly, none of the participating growers adopted this strategy (Fig. 3). Neonicotinoids at transplant were always followed by likely unnecessary foliar insecticide applications.

We were also interested by the degree to which pyrethroids dominated insecticide use in this crop, representing the top three most commonly used—and four of the top five—active ingredients. Because pyrethroids are incredibly cheap compared with other products, averaging as little as one dollar per acre for each application, there was a negative relationship between insecticide price and application frequency (i.e., growers preferentially applied the cheapest insecticide available); this trend appeared to be largely driven by the pyrethroids (Fig. 4). We suspect the high management intensity observed in watermelon is, in large part, facilitated by the extremely low cost of these products, which can be added as a form of inexpensive insurance, even if the insect pest pressure is low to nonexistent. These insurance sprays are likely further expedited by the susceptibility of cucurbits to foliar pathogens, resulting in regular fungicide applications to control diseases such as powdery mildew. Anecdotal observations suggest that insecticide/fungicide tank mixes are popular, allowing growers to ‘piggyback’ insecticides onto their fungicide regime without a separate field pass, making a cheap insecticide even cheaper. This introduces the potential for increased insecticide use due to tank mixes. In addition, there is growing evidence that fungicides can have synergistic effects when combined with insecticides. This synergism has been shown to increase bee mortality when compared to either the insecticide or fungicide on their own (Pilling and Jepson 1993, Biddinger et al. 2013, Sgolastra et al. 2017). These effects mean that prophylactic tank mixes could increase the risk of contact and toxicity for nontarget insects such as pollinators.

Last, we found that field size was a strong predictive factor determining the amount that growers invest in insecticide-based management, with larger producers spending ca. 10 times more per acre than small producers (Fig. 5). This increase was a function of large farms both having higher numbers of insecticide applications per field (i.e., they spray more often) and, on average, using more expensive products than smaller-scale growers. Surprisingly little data are available on the influence of farm size on insecticide use behaviors. However, several factors could explain this pattern. Their comparatively large economic investment in one commodity likely selects for a more conservative approach, compared with smaller growers where risk is spread among numerous crops/pests. Alternatively, large farms may be more profitable, resulting in more expendable income to invest in inputs such as insecticides. Large farms may also receive discounts for purchasing bulk quantities that would not be available to small farms purchasing smaller quantities. Any bulk discounts that large farms receive could make individual applications cheaper but are unlikely to impact our broader economic conclusions.

Finally, it is worth noting that field size differences in insecticide use are critical for identifying the types of farms to target for minimizing nontarget effects. All else being equal, large farms are more ecologically influential on the greater ecosystem simply due to occupying more land area. If these farms are also more likely to apply insecticides, which our data strongly indicate, then extension efforts aimed at implementing IPM programs would be best served in targeting these producers.

### Economics of IPM

We calculate an average savings of $195 from reducing one insecticide application for an average sized field (20 acres) using an average cost insecticide ($9.75/acre). Extrapolating over the seasonal average of five applications per field results in ca. $1,000 for insecticide inputs that would be saved if pests never reached threshold and thus were never treated. However, this value obviously scales with field size. One of our largest fields (100 acres), for example, was also the most intensively managed with 10 insecticide applications over the season, resulting in an overall cost of $14,036. In comparison, the cost of scouting ranged from $29 to $120, depending on speed and pay rate, which means that eliminating just one insecticide application will result in a net savings under most circumstances. The cost of the application itself was not included due to the frequency of tank-mix insecticide and fungicide applications. Any eliminated insecticide applications that were not part of a tank-mix treatment would elicit even greater savings. These conclusions correspond with other studies in watermelon that found pest monitoring using IPM is more cost effective than weekly sprays with savings of $90–$139 per acre (Lima et al. 2014).

A limitation of our study is that we do not have crop production data. Ideally, these would be used to calculate cost savings relative to yield comparisons across pest management systems. Yet, most studies have reported that insecticides do not impact watermelon yield (Lu et al. 2003, Dogramaci et al. 2004, Lima et al. 2014) and thus, we do not expect production differences across the insecticide gradient. In fact, prior research in cantaloupe concluded that IPM increases yield compared to prophylactic insecticide management due to interference with pollinators (Brust et al. 1996, Brust and Foster 1999), potentially making IPM even more cost-effective than the above calculations suggest (but see Foster and Brust 1995).

A major unanswered question remaining is the degree to which cost savings impact the decision-making process employed by growers. In many cases, other factors such as avoiding the evolution of resistance in pest populations is a far more influential factor in IPM adoption (Leach et al. 2019). To our knowledge, resistance has not been documented for SCB to the active ingredients deployed here. Future research efforts will aim to establish whether the calculated amount of money saved is sufficient to cause behavioral shifts in pest management practices and uncover additional factors (e.g., declines in pollinator health or function) that drive IPM adoption in watermelon and related cucurbits.

## Supplementary Material

Supplementary data are available at *Journal of Economic Entomology* online.

Supplementary Appendix 1. Economic and intensity of management assessment for each field. Insecticide cost per acre was calculated with the price per fluid ounce of insecticide multiplied by the per acre application rate for all insecticides applied across the season. Intensity of management was assessed with two approaches, the presence or absence of a prophylactic pretreatment and the number of insecticide applications across the watermelon growing season. In addition to these approaches, individual active ingredients used and the number of applications of each is included. The table is sorted by the presence of a pretreatment and then in descending order of the number of insecticide applications. 

toz346_suppl_Supplementary-Appendix-1
