# Supplementary material for: Comparing Prophylactic Versus Threshold-Based Insecticide Programs for Striped Cucumber Beetle (Coleoptera: Chrysomelidae) Management in Watermelon
Source: J Econ Entomol. 2020 Jan 5;113(2):872–81. doi: 10.1093/jee/toz346 (PMC7136195; doi:10.1093/jee/toz346)
Supplement: toz346_suppl_Supplementary-Appendix-1 [file toz346_suppl_supplementary-appendix-1.pdf]

Digital Appendix 1: Economic and intensity of management assessment for each field. Insecticide cost per acre was calculated with the price per fluid ounce of insecticide multiplied by the per acre application rate for all insecticides applied across the season. Intensity of management was assessed with two approaches, the presence or absence of a prophylactic pre-treatment and the number of insecticide applications across the watermelon growing season. In addition to these approaches, individual active ingredients used and the number of applications of each is included. The table is sorted by the presence of a pre-treatment and then in descending order of the number of insecticide applications.

| Pre-Treatment | # of Insecticide Applications | Insecticide Applications of Chemicals | Field | Year | Insecticide Cost per Acre |
|---|---|---|---|---|---|
| Yes | 10 | Bifenthrin 3x, Permethrin 2x, Spiromesifen, Chlorantraniliprole 2x, Fenpyroximate, Cyantraniliprole | 8 | 2017 | $140.36 |
| Yes | 9 | Imidacloprid (Drench), Chlorantraniliprole 2x, Spiromesifen, Permethrin 2x, Bifenthrin 2x, Abamectin | 8 | 2018 | $143.43 |
| Yes | 9 | Thiamethoxam (Tray Treatment), Lambda-Cyhalothrin 4x, Spiromesifen 2x, Acetamiprid, Fenpyroximate | 11 | 2018 | $142.81 |
| Yes | 6 | Imidacloprid (Drench), Cyfluthrin 2x, Lambda-Cyhalothrin 2x, Abamectin | 6 | 2017 | $46.30 |
| Yes | 5 | Thiamethoxam (Tray Treatment), Lambda-Cyhalothrin 2x, Spiromesifen, Fenpyroximate | 11 | 2017 | $71.48 |
| Yes | 4 | Thiamethoxam (Drench), Spiromesifen, Permethrin 2x | 13 | 2017 | $42.32 |

| Pre-Treatment | # of Insecticide Applications | Insecticide Applications of Chemicals | Field | Year | Insecticide Cost per Acre |
|---|---|---|---|---|---|
| Yes | 4 | Thiamethoxam (Drench), Spiromesifen, Lambda-Cyhalothrin, Acetamiprid | 13 | 2018 | $72.42 |
| Yes | 3 | Imidacloprid (Drench), Lambda-Cyhalothrin 2x | 4 | 2017 | $26.47 |
| Yes | 2 | Imidacloprid (Drench), Lambda-Cyhalothrin | 4 | 2018 | $21.53 |
| No | 9 | Lambda-Cyhalothrin 2x, Cyfluthrin 4x, Permethrin 2x, Dimethoate | 1 | 2017 | $39.81 |
| No | 8 | Cyfluthrin 3x, Spiromesifen 2x, Abamectin, Flubendiamide, Acetamiprid | 10 | 2017 | $124.33 |
| No | 8 | Bifenthrin 8x | 7 | 2017 | $14.40 |
| No | 7 | Bifenthrin 7x | 7 | 2018 | $12.60 |
| No | 7 | Permethrin 7x | 12 | 2018 | $12.60 |
| No | 7 | Permethrin 7x | 12 | 2017 | $12.60 |
| No | 5 | Spiromesifen 2x, Cyfluthrin, Acetamiprid, Abamectin | 10 | 2018 | $109.46 |
| No | 4 | Abamectin 2x, Cyfluthrin, Acetamiprid | 16 | 2017 | $65.01 |
| No | 4 | Lambda-Cyhalothrin 3x, Bifenthrin | 6 | 2018 | $12.28 |
| No | 4 | Permethrin, Lambda-Cyhalothrin, Imidacloprid, Spiromesifen | 16 | 2017 | $36.38 |
| No | 3 | Permethrin 3x | 9 | 2018 | $5.40 |
| No | 3 | Permethrin 3x | 9 | 2017 | $5.40 |

| Pre-Treatment | # of Insecticide Applications | Insecticide Applications of Chemicals | Field | Year | Insecticide Cost per Acre |
|---|---|---|---|---|---|
| No | 3 | Abamectin, Acetamiprid, Flupyradifurone | 15 | 2017 | $61.07 |
| No | 2 | Abamectin, Bifenthrin | 15 | 2018 | $16.68 |
| No | 1 | Bifenthrin | 3 | 2018 | $4.62 |
| No | 0 | N/A | 2 | 2017 | $0 |
| No | 0 | N/A | 1 | 2018 | $0 |
| No | 0 | N/A | 5 | 2017 | $0 |
| No | 0 | N/A | 5 | 2018 | $0 |
